# Counseling Psychological Understanding and Considerations of the Metaverse: A Theoretical Review

**DOI:** 10.3390/healthcare11182490

**Published:** 2023-09-07

**Authors:** Kunho Lee

**Affiliations:** Department of Counseling Psychology, College of Health and Welfare, Sahmyook University, Seoul 01795, Republic of Korea; leekunho@syu.ac.kr

**Keywords:** metaverse, counseling psychology, mental health, mental disorder, virtual reality, healthcare, digital experience

## Abstract

The COVID-19 pandemic has triggered the interest in and demand for online platforms that can replace traditional face-to-face activities. Accordingly, metaverses have been increasingly used across society, especially in the Mental health field. This broad use of metaverses is now recognized as a major trend that will drive various industries across healthcare and society. In response to this societal change, this study provides a theoretical framework for understanding and applying metaverses as therapeutic spaces in mental health fields through a conceptualization and characterization of metaverses for a range of technologies and services. The value of metaverses as a human-centered “field of experience” was identified and proposed based on the essential aspect of the user, the human being, rather than from the technology used. According to existing psychotherapeutic theories, four therapeutic concepts for the metaverse were proposed: metaverse as an “average expectable environment” in the developmental and therapeutic categories of the self, a transitional stage for adaptation to reality, a creative space for memory and communication for the self, and an expanded or “surplus” reality. This work is expected to be a useful basis for expanding new psychotherapeutic strategies and methods as therapeutic spaces for maintaining mental health through the metaverse.

## 1. Introduction

Although we have reached the tail end of the global health emergency caused by the COVID-19 outbreak [1], the pandemic itself has drastically changed our lives. In particular, the shift toward a non-face-to-face environment has increased the demand for online platforms that can replace traditional face-to-face activities, and the rapid development of technology has increased the popularity and use of the metaverse in various fields [2]. In the past, the metaverse was a narrow concept that appeared mainly in games and movies; however, in recent years, it has developed into a broad concept of reality that combines online and offline elements and is rapidly expanding into various fields, such as education, healthcare, business, and culture [3]. The global metaverse market is expected to reach USD 679 billion by 2030 [4], indicating that future growth and development will be driven by metaverses in various fields. Additionally, the value of the metaverse in providing consultancy services related to human psychology and mental health is expected to increase significantly. A study by J.D. Power published in 2022 supports this need, with more than half of the respondents (67%) preferring online therapy to face-to-face therapy and 94% indicating that they will continue to use such services for their healthcare needs in the future [5]. Notably, the number of studies using metaverses to treat eating disorders, autism, attention deficit hyperactivity disorder, anxiety, phobias, and post-traumatic stress disorder continues to grow [6,7].

Given the social and contextual dimensions described previously, the understanding and applicability of a counseling psychology approach to the metaverse requires further discussion. In particular, the concepts and characteristics of a broad range of technologies and services can be discussed from a counseling psychology perspective to draw a macroscopic terrain for the use of the metaverse in clinical practice and to provide a basis for further research. Evidently, concerns and uncertainties remain with regard to the limitations of the applicability of new technologies and their potential impact on mental health based on existing counseling psychology theories and principles [8]. Nevertheless, we must recognize that we are already living in a new era in which our daily lives cannot be considered separate from the world of technology, just as advanced technologies can only be popularized when they acquire a social context through human interactions [9]. Therefore, the current study aims to conceptualize and understand the metaverse from a counseling psychology perspective and examine its applicability. Accordingly, this study comprehensively analyzes the general concept, types, and development of the metaverse to identify its psychological characteristics and significance and to provide a theoretical basis for understanding and applying them.

## 2. Background Review

### 2.1. Conceptual Understanding of the Metaverse

While “metaverse” was one of the most popular tech terms in Google Trends in 2021 and has been perceived as a new term because of the global pandemic, it is not actually a new concept [7]. The metaverse has gained considerable attention during the recent pandemic, but it has long been widely studied; in particular, the basic elements related to virtual reality (VR) have been studied for more than 50 years in terms of limited immersion and interaction [10]. Despite the differences in terminologies, the metaverse is regarded as the sum of the Internet, existing online-related technologies, and various VR technologies. It is also considered as the most concentrated online technology in recent years.

The term “metaverse” was first introduced in Neal Stephenson’s 1992 science fiction novel Snow Crash. The word is a combination of the Greek words “meta”, meaning transcendence or transformation; and “universe”, meaning the universe and the world of human experience [11]. It is described as a vast virtual environment that coexists with the physical world and in which people interact through digital avatars. The discussion of the metaverse began in earnest with the advent of Second Life, launched in 2003 by the American game developer Linden Lab (San Francisco, CA, USA) [12]. In 2007, the Acceleration Studies Foundation (ASF), a U.S. nonprofit organization, proposed in its Metaverse Roadmap that the metaverse should not be viewed as a virtual space but as a connection between physical and virtual worlds [13]. Davis et al. defined the metaverse as an immersive three-dimensional virtual world that resembles the real world but has no physical boundaries, where people interact with other users and software agents through avatars [14]. Although a clear consensus has yet to be reached with regard to the definition of the metaverse because of the broad range of complex technologies and services involved [13], we can summarize its various existing definitions as follows: (1) a three-dimensional (3D) shared virtual space, (2) the use of avatars and the continuity and synchronization of identities through them, and (3) immersion through interactions and interoperability [15].

The conceptual definition of metaverse will continue to evolve into a more expansive and advanced notion based on the development of related technologies through the construction and use of metaverse platforms [16], which will fundamentally change the way humans interact with the digital world in the future.

### 2.2. Distinguishing Metaverse Types

**Figure 1:**
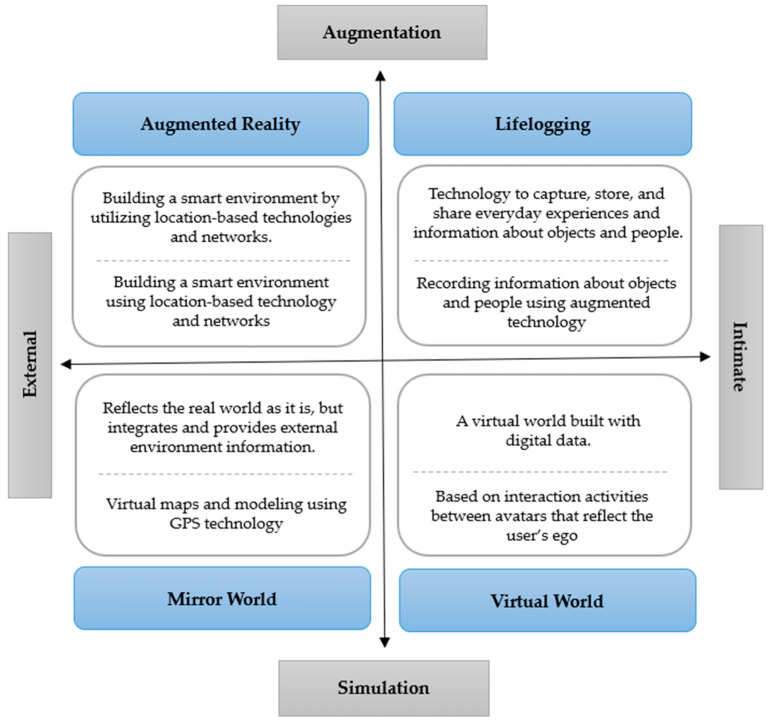
Types of metaverse [13,25].

For a deep understanding of metaverse spaces, they must be categorized according to their embodied forms. In its Metaverse Roadmap, the ASF has categorized the metaverse into four main types [13]: augmented reality (AR), lifelogging, mirror world, and virtual world. This classification is based on the form of the embodied space and information. As shown in Figure 1, the horizontal axis that separates the relationship between the technology and the user, the private/intimate domain on the right, focuses on the user’s identity and expression of behavior while the external domain, which represents the relationship between technology and reality, focuses on information and control over the external world surrounding the user. In addition, augmentation at the top of the vertical axis emphasizes AR technology, which combines a layer of computer-generated images with the user’s environment. Meanwhile, simulation at the bottom focuses on technology that provides a virtual world that mimics reality as a place for interaction between users and objects. The four metaverse types are further described as follows:

1. AR supplements the real world with mixed reality and is created by overlaying virtual objects or interfaces on a physical environment that users recognize in their daily lives [17]. Pokémon Go, which gained popularity in 2016, and Apple’s ARKit are examples of AR. It is recognized as a form of technology that uses network information, location-aware systems, and interfaces to extend the actual physical world outside the individual [18].
2. Lifelogging is an extension of the inner world and refers to a space where users automatically record and disclose their experiences through smart devices and communicate with other users regardless of place and time [19]. The popular social media platforms Facebook and Instagram are typical examples.
3. The mirror world refers to a virtual space that reproduces the physical world as realistically as possible and adds additional information to it, modeling the appearance and content of the physical world around us as if it were reflected in a mirror [13,20]. Representative examples include Google Earth, Google Maps, and Airbnb.
4. The virtual world is a space for interaction between users and objects and is a fully immersive system that does not consider the physical reality environment. It is a digital world that uses 3D graphics and avatars as tools to reflect the user’s self [21,22]. Some of the most prominent examples include Roblox, Minecraft, and Second Life, which are the most popular gaming platforms among young people.

Other examples include mixed reality (MR), an environment in which real-world and virtual-world objects are presented together in a single display [23]; and extended reality (XR), a technology that represents the convergence of all existing realities, including AR, VR, and MR, which are implemented through a wide range of hardware and software [24]. 

There are still limitations in interpreting the existing technologies mentioned above as metaverse itself, but what is clear is that the presented metaverse types are evolving and showing a tendency toward convergence, where their boundaries become increasingly blurred [13]. In the future, they are expected to be understood as equivalent to reality and as primary concepts that fuse and interact with reality rather than as secondary concepts that fill in gaps in the real world.

### 2.3. Applications and Prospects of the Metaverse in Counseling Psychology and Mental Health

The pandemic has forced us “to go virtual” and has led to the rapid development of metaverse-related technologies. This trend has increased the interest in not only the metaverse and its use in many fields, including healthcare and mental health, but also its potential for future growth [21]. For example, prior to the COVID-19 pandemic, only 43% of U.S. healthcare facilities were able to provide telemedicine services using metaverse-related technologies; however, by 2020, when COVID-19 became a pandemic, this number had risen dramatically to 95%, lending credence to this claim [26]. In particular, the use of metaverses in counseling and mental health deserves further attention. The metaverse, which has been considered the most integrated online technology in recent years, is the sum of the Internet, online-related technologies, and various VR technologies. Research and treatment using various metaverse technologies, such as AR and VR, are actively being conducted in the clinical field [10,21], and the value of the metaverse in the medical context, or “MEDverse” [6], is expected to increase in the future [27].

Among the characteristics of metaverses, immersion and connection to the real physical world through interactions and operations in virtual shared spaces are recognized as key components for healthcare in metaverse spaces [6,28,29]. Specifically, metaverse-related technologies, such as realistic 3D graphics and haptic and interoceptive technologies, can create interactive first-person experiences that are more ecologically valid than traditional one-way experimental stimuli and can elicit physical and psychological responses in users that are similar to those in the real world [30,31]. Some examples of metaverse-related technology applications such as AR, and VR in counseling and clinical therapy, are discussed below:

1. Addictions and eating disorders: The safe and controlled VR environment provides a setting in which individuals can be exposed to a variety of stimuli (objects or situations), assess which stimuli trigger the highest levels of craving, and learn how to cope with these stimuli [10,32]. It can also help individuals with eating disorders improve their satisfaction with their body and appearance by acquiring new knowledge about the body, learning how to recognize their body, and disengaging from emotional experiences [33].
2. Anxiety and phobias: Virtual reality exposure therapy (VRET) helps to treat anxiety and certain phobias through simulation technology that allows precise contact with fear-inducing stimuli through visual and auditory sensory channels [34,35]. More recently, AR exposure therapy has also been used to treat anxiety and phobias given its better sense of realism and reality judgment than VRET [36].
3. Post-traumatic stress disorder: As part of exposure therapy, a safe virtual environment is provided to help clients access and confront their traumatic memories, not merely using their imagination, to learn and develop appropriate responses and coping mechanisms [37,38].
4. Paranoia and attention deficit hyperactivity disorder: An objective assessment of symptoms can be achieved by observing clients’ reactions to situations or objects in a hypothetical environment [39,40]. Meanwhile, teaching and practicing new coping behaviors can help manage symptoms and increase adjustment to daily life.
5. Autism spectrum disorder: AR and VR environments can be used to safely induce new interpersonal skills and abilities through easily controlled therapeutic environments [41]. They enable people with autism spectrum disorder to practice real-world social skills without worry or anxiety about making mistakes or being rejected, which they often experience in real life. In addition, cognitive therapy using VR has been shown to be effective in improving social skills, concentration, cognition, and memory [35].
6. Stress and pain management for mental health: Immersive virtual spaces can help individuals manage their stress and mental health by serving as a venue to practice mindfulness, meditation, or yoga [42,43,44]. By providing a simple form of distraction, VR scenarios can reduce perceived stress and pain [7]. Studies have shown that these methods are more effective than traditional therapies in managing depression, anxiety, fatigue, and pain [45].

For better understanding, Table 1 summarizes some research examples that utilize metaverse-related technologies such as VR.

In discussing psychotherapeutic techniques based on the metaverse, it is necessary to recognize the various advantages that can be obtained through the metaverse environment as well as the possibility of withdrawal. Psychotherapy solutions using the metaverse have different psychological reactions for each individual user, so the possibility of a drop in retention rate and dropout is inevitable. Variables such as technical motivation, developmental stage, and sociodemographic context should be considered along with non-verbal signals or interpretation problems of real-time responses [46]. Nonetheless, as the metaverse of modern technology continues to grow and advance with the rapid development of related technologies, new methods and technologies for treating mental health disorders are expected to evolve steadily. In particular, the recent emergence of generative AI, which is changing the social and economic landscape, is expected to be another game-changer that will transform the existing metaverse ecosystem. With the advent of generative AI, the metaverse, which has been attracting attention for a while, may seem to have lost its presence, but recently, generative AI is hotly emerging as a major driving force for the change and growth of the metaverse [47,48]. This is interpreted as the fact that generative AI has made it possible to create an environment in which the metaverse can be produced more easily and quickly. For example, as generative AI creates an environment in which anyone can easily and quickly create various virtual humans, it is possible to implement interactions with avatars and NPCs (non-player characters), which is expected to change the ways of interaction in the metaverse. These technologies are becoming increasingly applicable as therapeutic-mediated tools, such as psychotherapy and education, in the metaverse as a therapeutic space [49], which raises expectations for the evolution and development of the metaverse as a therapeutic space.

### 2.4. Approaching and Understanding the Metaverse in Counseling Psychology

In light of the ongoing technological therapeutic revolution, the importance of each individual’s attitude and theoretical and philosophical stance toward the metaverse is becoming increasingly important in counseling and mental health. Understanding the metaverse from a counseling psychology perspective before the mere functional use of technology is timely, albeit slightly late, relative to the development and use of technology. Such effort is significant because it allows us to evaluate and reflect on reality and participate in the development of theoretical foundations and in decisions about the development and use of technology. Furthermore, such discussions are essential because they provide a foundation for the appropriate use of metaverses in the field of counseling psychology and a deeper understanding of their social value.

The definition and categorization of the metaverse discussed herein can serve as a good starting point for understanding new concepts of the metaverse. However, in terms of understanding the objects based on the technology used, we overlook the fact that humans use this space. The physical world that we are currently a part of is made up of many materials, but these materials are only available to humans who enjoy this space and not for the purpose of the world itself. Similarly, our understanding of the metaverse should focus on humans rather than on technology. Practical discussions of the metaverse should focus on the experiential aspects of the metaverse based on humans rather than on the technological and physical aspects of virtual worlds that are often used in the existing definitions and typologies of metaverses [50]. While technological change is important, the current discussion of the metaverse has been triggered by the demands of living with, and even being replaced by, the metaverse. Hence, a human-centered counseling psychology approach and understanding of the metaverse is even more relevant, and in this context, the metaverse has value as a human-centered “field of experience”. 

First, the metaverse can be regarded as an “average expectable environment” in the developmental and therapeutic categories of the self. The term “average expectable environment” was proposed by Hartmann and refers to the environmental conditions that must be provided for the independent development of the self [51]. It is derived from the theory of ego psychology, which focuses on the problem of adaptation of the self within the interrelationship between the organism and its environment. The term can also be partly regarded as an application of the theory of ego psychology to today’s conceptual perception of the metaverse. From the perspective of ego psychology, humans are understood in terms of adaptation, reality testing, and defense [51]. Self-psychology involves understanding how these human capacities regulate and deal with the needs, emotions, and fantasies of the human psyche and the demands of the real world [52]. The metaverse can be perceived as a therapeutic space that can provide an “average expectable environment” that supports the growth and adaptation of the self to escape ego deficit, an abnormal developmental state caused by the lack or failure of the ego functions of adaptation, reality verification, and defense, through the artificial implementation of various client-centered optimized therapeutic environments. This applicability reminds us of an active need to continue conducting research on the implementation of various counseling psychology theories in a metaverse environment, beyond existing face-to-face counseling techniques, using metaverse-related technologies. 

Second, the metaverse can be understood from a counseling psychology perspective as a “transitional stage” for adjustment to reality. This term is derived from the term “transitional object” in object relations theory in psychotherapy and can be understood as a bridge between a person’s inner needs and the outer reality [53]. Such a notion can provide spatial meaning to the therapeutic space of the metaverse as a “facilitating environment” that can serve as a sanctuary for the eternal work of human beings and as a transitional space of experience in which their inner and outer lives are co-created. The safe and immersive counseling environment provided by the metaverse is well suited as a therapeutic environment for modifying and correcting the inner object world that distorts the real world. This feature will allow clients to integrate their internal object relations that are not integrated into the fabric of their personalities and maximize their personal growth. 

Third, the metaverse is a semantic space that captures the value of life as data and can be perceived as a creative space of memory and communication for the self. Among the types of metaverses, lifelogging is defined by the ASF as an accessible archive that digitally records a user’s life [13]. Here, the archive refers to memories, observations, and daily records of objects and people and the communication and behavior created through the user’s experiences. Lifelogging in the metaverse is of therapeutic interest as a creative space of memory and communication for the self because the recorded information has practical value that can be used at any point in time for therapeutic purposes. The past experiences and life histories of clients are recorded as data and can be recreated and communicated at any time for therapeutic purposes, thus creating the possibility for inner growth and change. For example, in the treatment of stress, phobias, PTSD, and anxiety, digitally recorded and accessible life records can be recognized as a very useful therapeutic value in that they enable the reproduction and re-recognition of problematic memories and situations. This could have therapeutic value in that it could provide a framework for step-by-step recreation and objectification of a person’s digitally recorded life in the therapeutic phase due to cognitive distortions caused by the client’s bad memories and various avoidance reasons. In a similar way, recent applications of VR to treat depression and improve cognitive function in older people [54,55] and attempts to digitally reconstruct the dead as part of grief counseling therapy are worthy of further attention to understand and utilize the conceptual dimensions of this classification [56]. 

Finally, the metaverse allows for the experience of “surplus reality”, an expanded reality that transcends the categories of reality within the scope of therapy. The concept of surplus reality was proposed by Jacob Levy Moreno, the founder of drama therapy, and refers to an alternate reality in which emotions are purified and life is recreated by allowing the client to experience a life that exists within them but has not yet been lived [57]. Through surplus reality, clients have the opportunity to act out any pent-up emotions and thoughts that they have been unable to express or any repressed instinctual needs, dreams, and desires; they can also confront wounds, traumas, and situations that they wished to avoid in the past so that they can develop the strength to overcome problematic situations [58]. The metaverse facilitates the implementation of surplus reality, an extended reality for therapeutic purposes, using immersive media, such as haptic and interoceptive technologies where the human sensory experience is central. This feature increases its potential as an experiential space that can maximize therapeutic effects by overcoming physical limitations, such as the stage, audience (group), and auxiliary ego [59,60] that constitute existing drama therapy except for the client, and by providing various realistic situations and scenarios without constraints, such as participants and the environment. 

The concepts of metaverse as a therapeutic space discussed above are summarized in Table 2.

### 2.5. Considerations and Suggestions for Therapeutic Applications of the Metaverse in Counseling Psychology

Given the potential value of the rapidly expanding and evolving metaverse environment in various fields, its application in psychological counseling is promising. However, several potential risks and side effects have been predicted, and such potential risks associated with the application of new technologies in the field of counseling psychology for mental health must be anticipated and identified. First, privacy and data security vulnerabilities exist in the metaverse, and they need to be addressed and further explored [21,61]. In particular, medical records generated through therapeutic relationships and clients’ basic personal information require special attention. This issue is related to trust, which is at the core of forming a therapeutic alliance with clients and can be recognized as the most integral ethical responsibility in the therapeutic phase. In addition, information dysfunction, such as hacking, personal information leakage, and invasion of privacy in the cyber environment, is gradually worsening; therefore, technical efforts and research to create and maintain a safe therapeutic environment in the metaverse will continue to be a challenge in the future. Various regulations and laws should also be developed to keep pace with the rapid changes in the development and use of metaverse-related technologies. Second, the fact that the use of the metaverse for therapeutic purposes may lead to other mental health problems should be recognized [62]. The use of the metaverse for mental health has several therapeutic benefits and potential risks. Indeed, the excessive use of digital technologies is not limited to physical symptoms such as vertigo as it also poses risks that cause other mental health problems, such as paranoid thoughts, addiction, depression, and anxiety [7,21,63]. Therefore, the research and evaluation of potential mental health harms and alternatives, as well as the multiple benefits of using the metaverse in the therapeutic domain, should be encouraged. Third, extensive attention needs to be paid to clients’ experiences with counseling and psychotherapy interventions in the metaverse. Although the context of therapeutic interventions in the metaverse is different, in cyber-counseling over the Internet, 9.3% of the participants reported experiencing at least one adverse effect [64], especially because some of the symptoms worsened during treatment. In the case of therapeutic interventions in the metaverse, developers and counselors should pay close attention to clients’ experience with the treatment program they provide as stress could be aggravated by not only the difficulties in understanding the treatment and using the device from the patient’s perspective, but also the physical side effects [65]. Also, human-centered design in terms of the mental health system is a major factor that needs to be carefully considered. Establishing a user-centered system environment as a powerful empirical method to integrate practitioners and participants who operate treatment programs and to provide quality treatment with guaranteed effectiveness to all who are participating in the treatment is also a task to be solved together [63,66]. Fourth, multidisciplinary convergence and collaboration are essential for the therapeutic application and utilization of the metaverse in the field of counseling psychology. The metaverse refers to the totality of various technologies, such as information and communication technology and VR technologies. Therefore, the ways to use it in counseling and psychotherapy interventions should be actively explored through multidisciplinary collaboration. Its effectiveness should also be scientifically verified so that it can be firmly established as an evidence-based counseling and treatment technique. In order to realize this, it would be a good example to establish a system that can work together, centered on related organizations or government departments, to improve and maintain related laws and systems to form a multidisciplinary convergence and cooperative environment [67]. Furthermore, the therapeutic limitations and side effects of using the metaverse, as well as the related ethical issues, should be discussed in greater depth. Recent reports of sexual abuse in the metaverse have raised serious concerns [68]. The immersion and connection to the real physical world that characterizes the metaverse require further attention as virtual spaces elicit the same physical and psychological responses as experiences in the real world [30,31,69]. Finally, while metaverses can expand access to care for remote and marginalized populations, blind spots in care caused by constraints such as cost and equipment may exist and should thus be recognized. In addition, older people may be unfamiliar with the use of equipment. Therefore, alternative methods should be considered, and further research in this area is suggested. 

## 3. Conclusions

We live in a world that no one could have predicted. Just as our reliance on personal computers has become a natural part of our lives, as we move into the new realm of mobile devices, the metaverse will become a new paradigm that will fundamentally change the way we interact with the digital world. Especially for the digital native generation, who are currently recognized as the main users of the metaverse, the future of the metaverse will be a natural place to live, not only as a natural space for daily life, but also as a place where economic activities can take place [70]. As Roy Amara once said, “We tend to overestimate the effect of a technology in the short run and underestimate the effect in the long run”; thus, proper evaluation and preparation for the long term and beyond the short term are imperative [71]. This study discusses the concept and characteristics of the metaverse for a broad range of technologies and services from a counseling psychology perspective and suggests a theoretical basis for understanding and applying the metaverse as a therapeutic space. This proposal will be useful in expanding new psychotherapeutic strategies and methods as a therapeutic space for maintaining mental health. Based on these findings, the development and interdisciplinary research of new therapies that utilize metaverses should be pursued.

This study can provide research value to the fact that it has newly established a theoretical basis for the metaverse as a therapeutic space through a counseling psychological approach based on human-centered understanding, not a technology-oriented approach. In particular, it is significant in that it not only provides a new understanding of the metaverse as a therapeutic space by linking the therapeutic concept of the metaverse based on existing psychotherapy theories, but also lays the theoretical foundation for expanding new psychotherapy strategies using it. The metaverse is a new concept that still lacks a unified definition, despite being a hot topic around the world [72]. For this reason, despite the fact that there are many studies using technologies such as VR and AR in the field of mental health, there are limitations in recognizing and understanding it as a metaverse itself. Therefore, establishing a theoretical foundation along with a conceptual understanding of the metaverse as a therapeutic space through the implementation of theoretical research methods such as this study is an essential task that must be preceded to conduct more research in the future. This will be more useful in expanding new psychotherapeutic strategies and methods as a therapeutic space for maintaining mental health, and based on this, development and convergence research on new therapies using the metaverse are expected. 

The following are the limitations of this study and suggestions for further research. First, this study is a theoretical review, and the results of this study are subject to the potential subjectivity of the researcher, which limits the generalizability of the findings. It is necessary to conduct a comprehensive literature review using databases for a more comprehensive review of existing studies. Second, although various studies related to metaverses have been conducted, studies in the field of counseling psychology are very scarce. Therefore, it is necessary to carry out meta-analysis studies that can derive comprehensive results by integrating various research data in the future. Finally, there is a limitation that the research related to the metaverse has not kept pace with the rapidly evolving technological expansion. Therefore, there is a need to respond more sensitively to related research topics, and follow-up studies such as metaverse treatment strategies and techniques applying generative AI, which has recently become an issue, should be followed.

## Figures and Tables

**Table 1:** Research cases applying VR, metaverse-related technologies in the field of counseling and psychotherapy.

Authors	Year of Issue	Title of the Article
Freedman, D. et al.	2017	Virtual reality in the assessment, understanding, and treatment of mental health disorders [10]
De Carvalho, M.R. et al.	2017	Virtual reality as a promising strategy in the assessment and treatment of bulimia nervosa and binge eating disorder: A systematic review [32]
Matamala-Gomez, M. et al.	2021	Virtual body ownership illusions for mental health: A narrative review [33]
Kim, S.; Kim, E.	2020	The use of virtual reality in psychiatry: A review [34]
Park, M.J. et al.	2019	A literature overview of Virtual Reality (VR) in treatment of psychiatric disorders: Recent advances and limitations [35]
Rothbaum, B.O. et al.	2010	Virtual reality exposure therapy for combat-related posttraumatic stress disorder [37]
Dellazizzo, L. et al.	2020	Evidence on virtual reality–based therapies for psychiatric disorders: Meta-review of meta-analyses [38]
Freeman, D. et al.	2010	Testing the continuum of delusional beliefs: An experimental study using virtual reality [39]
Fornells-Ambrojo, M. et al.	2015	How do people with persecutory delusions evaluate threat in a controlled social environment? A qualitative study using virtual reality [40]
Ahmadpour, N. et al.	2019	Virtual Reality interventions for acute and chronic pain management [42]
Navarro-Haro, M.V. et al.	2017	Meditation experts try Virtual Reality Mindfulness: A pilot study evaluation of the feasibility and acceptability of Virtual Reality to facilitate mindfulness practice in people attending a Mindfulness conference [44]
Ioannou, A et al.	2020	Virtual reality and symptoms management of anxiety, depression, fatigue, and pain: A systematic review [45]

**Table 2:** Understanding the metaverse as a therapeutic space from a counseling psychology perspective.

	Concept of Understanding
**M**	**M**emory and communication space for the self
**E**	**E**xtended reality as a “surplus reality”
**T**	**T**ransitional stage for adaption
**A**	**A**verage expectable environment for growth and development of the self

## Data Availability

Not applicable.
